# Synthesis and Evaluation of a Chitosan-Based Cationic Hydrogel with Strong Antifungal and Antibiofilm Activities Against Clinical Isolates of *Candida auris*

**DOI:** 10.3390/ph18040506

**Published:** 2025-03-31

**Authors:** Muhammad Kamran, Maryam Aftab, Afreenish Amir, Fatima Javed, Amtul Quddos Latif, Kausar Abbas Saldera, Abdul Ahad, Yousef A. Bin Jardan, Louise Ann Walker, Kiran Nisa, Faheem Ullah, Naseer Ali Shah

**Affiliations:** 1 Department of Biosciences, COMSATS University Islamabad, Islamabad 45520, Pakistan; kamranahmdani@gmail.com (M.K.); maryambiochem88@gmail.com (M.A.); kirannisa153@gmail.com (K.N.); 2 Department of Pathology, Rawalpindi Medical University, Rawalpindi 46000, Pakistan; afreenish.hassan@yahoo.com; 3 Department of Chemistry, Shaheed Benazir Bhutto Women University, Peshawar 25000, Pakistan; fatimajaved@ymail.com; 4 Jinnah Post Graduate Medical Centre, Karachi 77550, Pakistan; phdmolecularmedicine@gmail.com (A.Q.L.); k.salderabbas@gmail.com (K.A.S.); 5 Department of Pharmaceutics, College of Pharmacy, King Saud University, Riyadh 11451, Saudi Arabia; aahad@ksu.edu.sa (A.A.); ybinjardan@ksu.edu.sa (Y.A.B.J.); 6 School of Medicine, Medical Sciences and Nutrition, Institute of Medical Sciences, University of Aberdeen, Aberdeen AB25 2ZD, UK; louise.walker@abdn.ac.uk; 7 Bioresource Technology Division, School of Industrial Technology, Universiti Sains Malaysia, Gelugor 11800, Pulau Pinang, Malaysia

**Keywords:** *Candida auris*, antimicrobial resistance, antifungal activity, biofilm, candidemia, chitosan, calcofluor white, hydrogel

## Abstract

**Background: ***Candida auris* is a significant global health concern, due to its rapid transmission, high mortality rate, and resistance to commonly available antifungal drugs. **Methodology:** During the current study, a cationic polymeric hydrogel was developed using chitosan (CS), polyethylene glycol (PEG), and methacrylic acid (MAA). The respective solutions were mixed in a volumetric ratio of 2:1:1. After characterization, the hydrogel was assessed using antifungal, antibiofilm, and hemocompatibility assays. **Results:** The hydrodynamic radius of 554.7 ± 90.1 nm and zeta potential of 15.6 ± 1.09 mV indicate the ideal size and charge for topical applications and in vivo studies, respectively. The formulation exhibited improved thermal stability, enhanced swelling, and a drug release profile for non-Fickian diffusion. The hydrogel effectively inhibited fungal growth in agar plates (42 ± 7.31 mm zone of inhibition), with a mean IC_50_ of 15.17 ± 4.01 μg/mL and MIC of 29.30 ± 11.72 μg/mL. Calcofluor white (CFW) staining showed diffuse irregular yeast cells, suggesting increased membrane permeability, eventually leading to cell death. The hemocompatibility assay revealed no visible agglutination or hemolysis at the MIC value. The formulation exhibited significantly reduced biofilm formation compared to the growth control (*p* < 0.05). Additionally, in silico analysis revealed that MAA showed superior oral bioavailability, no inhibitory activity on cytochrome P450 enzymes, and low potential for toxicity through nuclear receptor signaling pathways. **Conclusions:** Cationic hydrogels show promise as potential antifungal treatments. The development of cost-effective and improved therapeutic methods is crucial to combat this deadly pathogen and to improve patient outcomes.

## 1. Introduction

*Candida auris*, a multidrug-resistant fungal pathogen, is a public health threat because of its high resistance to common antifungal drugs [1]. Its rapid global spread and severe systemic infections make it second on the priority list of critical pathogens created by the World Health Organization (WHO) [2]. The fungus persists in healthcare environments and is associated with nosocomial outbreaks [3]. Tertiary care hospitals are the primary source of over 50% of candidemia cases [4]. In the adult population, candidemia is associated with mortality rates ranging from 15% to 35% [5]. In neonates, the mortality rate attributed to candidemia is 10–15% [6]. After the first identification of *C. auris* in the ear canal of a patient in Japan in 2009, and the first reported invasive infection in South Korea in 2011, the fungus showed increased resistance to routinely used antifungal drugs [7]. According to the Centers for Disease Control and Prevention (CDC), 11,307 clinical cases of *C. auris* have been reported worldwide. The pathogen shows rapid transmission among immunocompromised individuals within healthcare facilities [8].

Outbreaks have been reported in different countries, including the United States, Spain, Canada, India, the United Kingdom, and Pakistan [9]. The fungus has emerged as a predominant pathogen in bloodstream infections, surpassing the prevalence of *Candida glabrata* and *Candida tropicalis* [7]. The virulence factors that contribute to *C. auris* infection are still under investigation. Different cell wall proteins, such as the agglutinin-like sequence (ALS1-7) and GPI-anchored protein (Pga1), facilitate adherence to host surfaces [10]. Factors such as the acquisition of nutrients [11], modulation of cell wall structure [12], utilization of two-component systems [13], and secretion of hydrolytic enzymes such as proteinases and phospholipases are involved in the pathogenicity of *C. auris* [14].

Both CS and PEG have been studied extensively for their potential use in drug delivery systems. CS is a natural biopolymer derived from chitin by deacetylation. The cationic nature of CS facilitates its binding to negatively charged fungal membranes, leading to increased membrane permeability and, eventually, death [15,16]. The copolymerization of CS with natural polymers serves as a network for the loading and sustained release of antifungal drugs [17]. The copolymerization of CS-PEG increases the stability of the loaded drug and reduces its toxicity [18]. Several researchers have studied the antifungal activity of hydrogels in the treatment of candidemia [19,20,21]. The CS-PEG drug delivery system has been used to deliver amphotericin B to resistant fungal strains [22]. CS-based hydrogels were found to be effective against vaginal candidiasis and co-infections [23]. Injectable hydrogels have potential for in vivo drug delivery in polymeric materials such as CS and PEG [24]. These hydrogels can be functionalized under the desired conditions and pH [25]. MAA has antifungal and antibacterial activities, and inhibits biofilm formation [26]. In view of global health threats and increased resistance to antifungal drugs, the current study aimed to establish novel therapeutic options. The antifungal activity of methacrylic acid interconnected in a CS-PEG network hydrogel was assessed against MDR *C. auris*. Low- and middle-income countries (LMICs), including Pakistan, face difficulties in identifying and treating these deadly pathogens. This study will pave the way for improved and cost-effective therapeutic methods that will ultimately improve the outcomes of candidemia patients.

## 2. Results

### 2.1. Culture, Identification, and ASFT of Isolates

Clinical isolates were cultured and identified using MALDI-TOF mass spectrometry. The breakdown of chromogenic substances in CHROMagar by β-N-acetyl hexosaminidase resulted in the formation of blue halo colonies. MALDI-TOF MS was used to confirm the identity of the isolates [27]. Antifungal susceptibility testing was performed on the four isolates. Of these, three isolates were sensitive to Fluconazole and Amphotericin B (ID: 1, 2, 3), whereas one isolate was resistant to both drugs (MDR) (ID: 4). All the isolates (100%) were sensitive to Caspofungin.

### 2.2. Synthesis of Hydrogel

**Figure 1:**
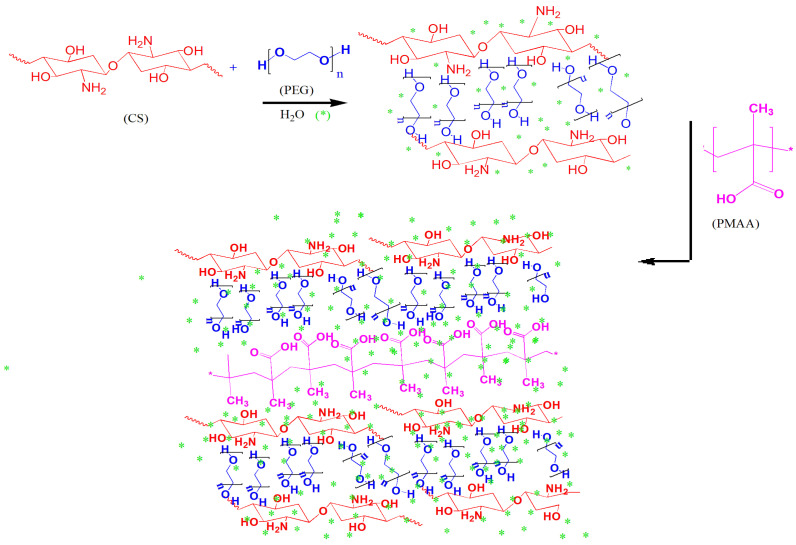
Synthetic scheme for preparation of hydrogels with hydrogen bonding between CS, PEG, and MAA. * represent the water molecules which overall strengthen the electrostatic interaction between oppositely charged molecules.

Hydrogel was synthesized using CS, PEG, and MAA. The hydroxyl groups of PEG initially form hydrogen bonds with the amino groups of CS, creating an initial structure. This hydrogen bonding stabilizes the hydrogel network through non-covalent interactions, in addition to the hydrogen bonding of CS and PEG with MAA, thus providing structural stability. The overall electrostatic interaction between oppositely charged species results in strong hydrogen bonding with the network structure, which is further strengthened by the water molecules; thus, the interfacial properties are enhanced, and the pendant groups are able to freely interact with each other and the cellular interfaces. The final hydrogel structure is a physically crosslinked network with CS chains interconnected by hydrogen bonding. These interactions result in a robust hydrogel network with physical crosslinking, making it suitable for various applications, such as drug delivery, wound healing, and tissue engineering. (Figure 1).

### 2.3. Zeta Size, Potential, and Polydispersity Index (PDI)

**Figure 2:**
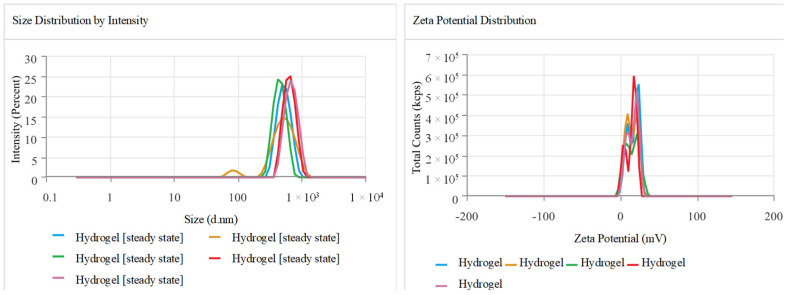
The peaks in the graph indicate a uniform size and ideal zeta potential for the drug delivery system. The readings were repeated using the instrument, highlighted by different colored lines.

The Z-average and polydispersity index (PDI) values of the hydrogel showed a moderate-to-strong degree of polydispersity in particle size, with a particle size of 554.7 ± 90.1 nm. The polydispersity index (PDI) was 0.308 ± 0.158, suggesting a narrow range of particle sizes. The zeta potential of 15.16 ± 1.097 mV suggests moderate stability and compatibility for in vivo systems. The conductivity of 1.694 mS/cm indicates a stable ionic environment, which could be beneficial for the hydrogel’s potential use in drug delivery systems (Figure 2).

### 2.4. Scanning Electron Microscope (SEM)

**Figure 3:**
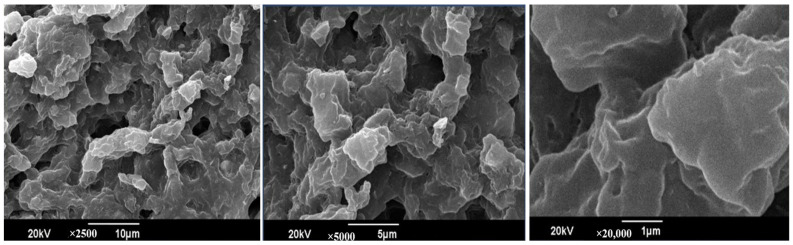
SEM micrographs of the CS-PEG network. Images were captured at magnifications of 10 µm, 5 µm, and 1 µm. This formulation exhibited a porous hydrogel network.

The SEM images show a porous, sponge-like structure of the CS-PEG-based hydrogel at different magnifications (10 µm, 5 µm, and 1 µm). The porous structure of the hydrogel shows dense interconnected CS-PEG. The open structure was formed due to different degrees of crosslinking between the amino group of CS and the hydroxyl group of PEG (Figure 3).

### 2.5. Fourier Transform Infrared (FT-IR) Spectroscopy

**Figure 4:**
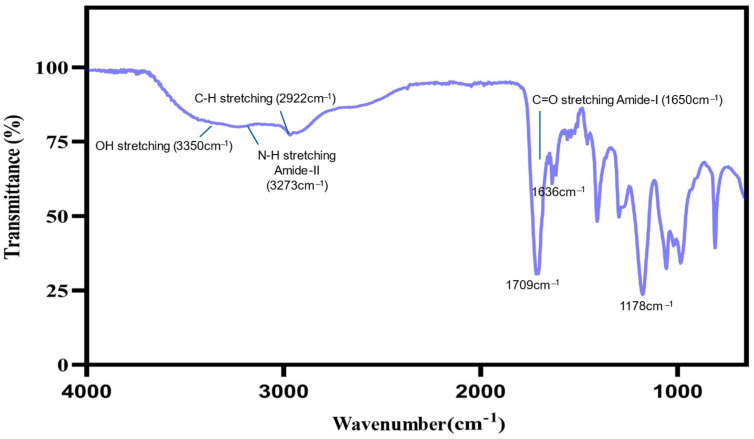
FT-IR spectrum of the hydrogel, displaying peaks indicating critical bonding between CS, PEG, and MAA.

The FTIR spectrum of the hydrogel prepared from CS, PEG, and MAA confirmed its successful formation. Key peaks in the spectrum include O-H stretching (3350 cm^−1^), N-H stretching (Amide-II, ~3273 cm^−1^), C-H stretching (2922 cm^−1^), C=O stretching (Amide-I, ~1650 cm^−1^), C=O stretching (~1636 cm^−1^), Carbonyl (C=O) stretching (~1709 cm^−1^), and C-O stretching (Amide-I, ~1178 cm^−1^). These peaks confirm the presence of functional groups from all three components, with amide bonds indicating the crosslinking and interaction of CS with the other components. The peaks corresponding to C=O stretching from MAA and CS confirm the integration of these materials into the hydrogel network [28,29,30] (Figure 4).

### 2.6. Thermogravimetric Analysis (TGA)

**Figure 5:**
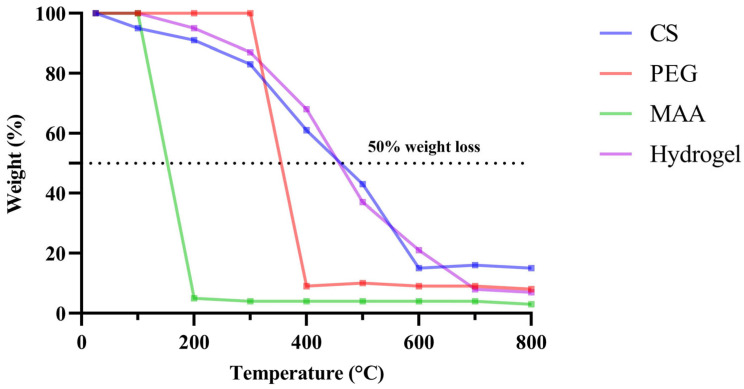
Percentage weight loss as a function of temperature. The individual components and formulations were subjected to a gradual increase in temperature to a maximum of 800 °C (10–20 °C/min). The instrument continuously measured and recorded the weight change of the sample as a function of the temperature.

Thermogravimetric analysis was conducted on the individual components and the formulation to examine the percentage weight loss as a function of temperature. Among the individual components, CS exhibited the highest stability at higher temperatures, whereas MAA degraded by almost 90% when the temperature exceeded 100 °C. With 50% weight loss observed at approximately 460 °C, the hydrogel demonstrated sufficient stability for sterilization requirements in biomedical applications. The experiment was conducted up to 800 °C, and even at this temperature, approximately 7% of the hydrogel weight remained (Figure 5).

### 2.7. Swelling and Degradation Assay of Hydrogel

**Figure 6:**
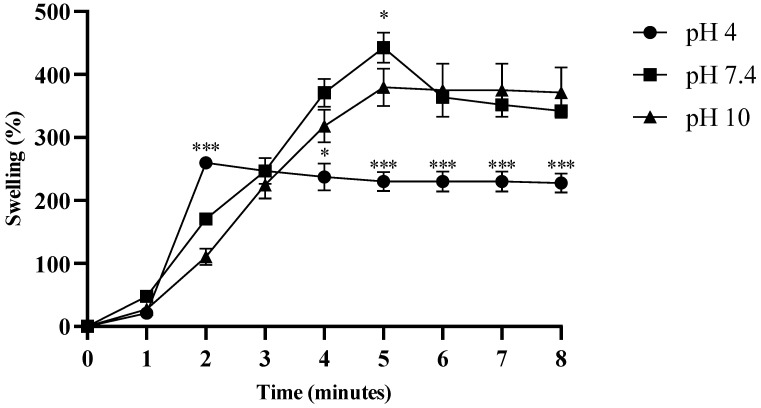
The swelling properties of the tested formulations. Swelling was performed at three different pH values: acetate buffer (pH 4.0), phosphate-buffered saline (pH 7.4), and borate buffer (pH 10). Significant differences were observed in hydrogel swelling at physiological (pH 7.4) and alkaline pH (pH 10), as compared to acidic pH (pH 4.0) (* *p* < 0.05, *** *p* < 0.0001).

**Figure 7:**
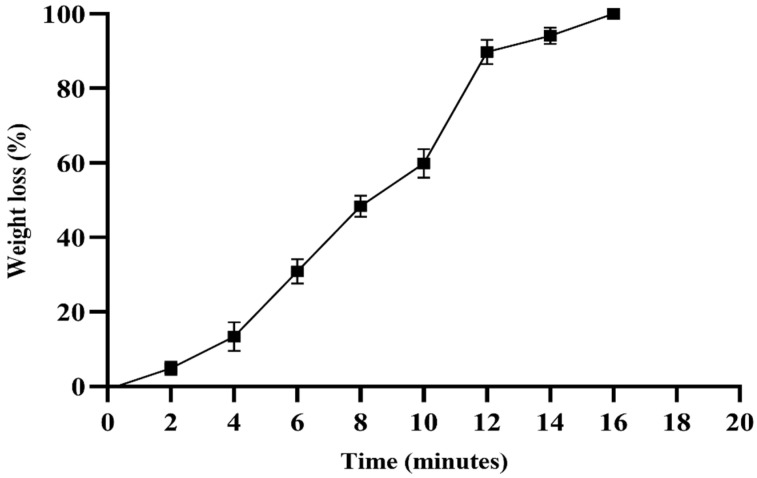
The degradation assay of the hydrogel was performed in PBS solution (pH 7.4). The percentage weight loss was calculated at different time intervals. The experiment was performed in triplicate.

The swelling properties of the hydrogel were evaluated at three different pH values (4.0, 7.4, and 10). At pH 4.0, the hydrogel exhibited a swelling capacity (>200%) after two minutes, and showed a significant difference at three minutes compared to at pH 7.4 and 10 (*p* < 0.0001). At pH 7.4, the hydrogel exhibited maximum swelling (>430%) after approximately 5 min, and exhibited a significant difference at pH 10 (*p* < 0.05). From five minutes onwards, the swelling was uniform at pH 4.0, and significantly different from the other two pH values (*p* < 0.0001) (Figure 6). The degradation assay was performed in PBS, and after maximum swelling (>430%) for approximately 5 min, the hydrogel began to degrade gradually, and lost 100% of its weight within 17 min (Figure 7).

### 2.8. Drug Entrapment Efficiency (%DEE) and Release Kinetics

**Figure 8:**
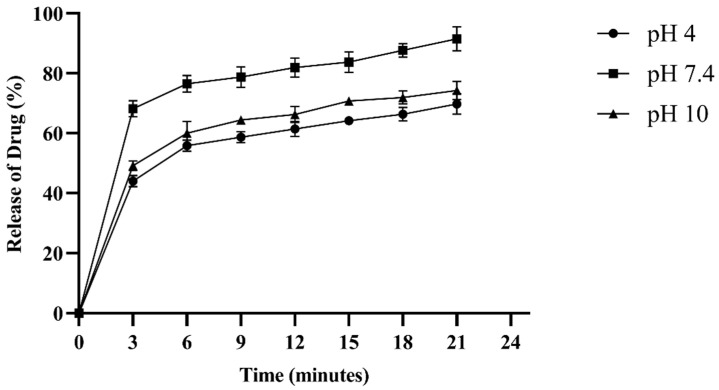
Fluconazole (32 µg/mL), an antifungal drug, was loaded into the hydrogel network to assess the drug entrapment efficiency (DEE%) of the formulation. The DEE% was measured at three different pH values. The experiment was performed in triplicate.

The drug entrapment efficiency of the hydrogel exhibited a rapid burst phase (0–3 min) at all pH values, with the fastest release occurring at physiological pH (pH 7.4). Subsequently, the stabilization phase (3–22 min) slowed down and stabilized the release rate. At pH 7.4, 91.25% of fluconazole was released in 21 min, whereas at pH 10, the release was 74.3%, and at pH 4, it was 69.8%. The rate of drug release was pH-dependent (Figure 8).

Table 1 presents the drug release kinetics and mechanisms at three different pH levels: acetate buffer (pH 4.0), phosphate-buffered saline (pH 7.4), and borate buffer (pH 10). The results indicate that the drug release rate was most consistent at pH 7.4, suggesting a stable release rate at physiological pH (pH 7.4). The Korsemeyer–Peppas model with R^2^ values close to 1 indicates a release mechanism of non-Fickian diffusion at all pH levels, involving a combination of diffusion and polymer relaxation. The release mechanism remained consistent across the different pH levels, suggesting the strength of the formulation under various conditions (Supplementary Figures S1 and S2).

### 2.9. Antifungal Activity of Hydrogel

**Figure 9:**
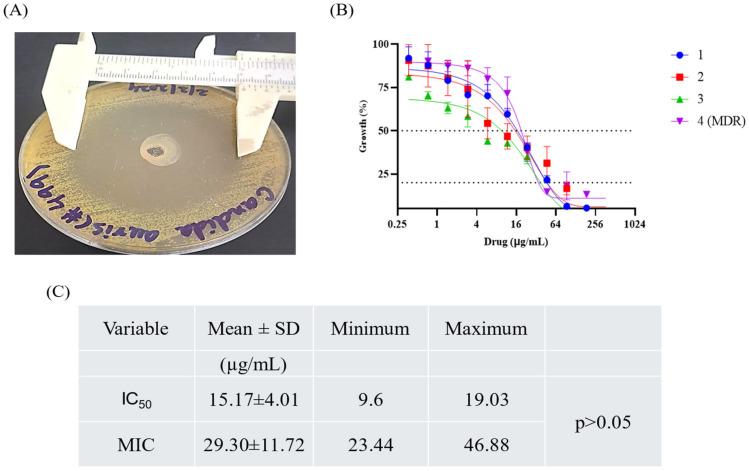
Different strains of *C. auris* were subjected to serial dilutions of the antifungal hydrogel. Zone of inhibition was measured (**A**) and IC_50_ values were calculated by fitting the dose–response curves to a sigmoidal model. Numbers 1, 2, 3, and 4 (MDR) represent the clinical isolates of *C. auris*. (**B**). MIC values were calculated according to the CLSI guidelines (M27, ED4). The MIC values were calculated in triplicates (**C**).

An agar plate was inoculated with *C. auris*, and the hydrogel was loaded into the well at the center of the dish. After 24 h of incubation, a 42 ± 7.31 mm zone of inhibition was measured (Figure 9A). The dose–response curves for various *C. auris* strains were measured at 600 nm after serial dilution of the hydrogel in a 96-well plate (Figure 9B). A decrease in the growth percentage was observed as the drug concentration increased, indicating that the hydrogel effectively inhibited fungal growth. The mean IC_50_ value was 15.17 ± 4.01 μg/mL, while the MIC value was 29.30 ± 11.72 μg/mL (Figure 9C). The images in Figure 9 demonstrate the efficacy of the hydrogel in combating different *C. auris* strains. No significant difference was found in the MIC and IC_50_ between MDR and sensitive isolates (*p* > 0.05).

### 2.10. CFW Staining and Fluorescent Microscopy of C. auris Cells Treated with Hydrogel

**Figure 10:**
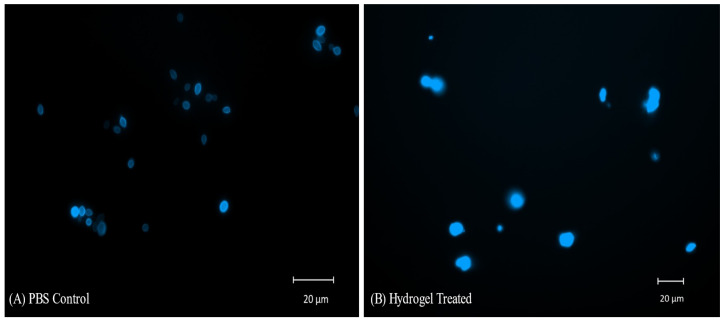
Yeast cells stained with CFW (20 µg/mL), showing a diffuse irregular appearance after hydrogel (30 µg/mL) treatment (**B**). CFW stained chitin in the cell wall and is clearly visible in the PBS control (**A**).

The images of hydrogel-treated yeast cells after staining with CFW show diffuse irregular yeast cells with improper morphology. The images suggest lysis, increased membrane permeability, and disintegration of the cell wall after the hydrogel treatment. Increased permeability leads to the staining of internal nuclear envelopes and polysaccharide-containing organelles, resulting in a diffused irregular appearance and higher absorption of fluorescence. The PBS control shows clear cell walls with oval-to-elliptical yeast cells (Figure 10).

### 2.11. Hemocompatibility Assay

**Figure 11:**
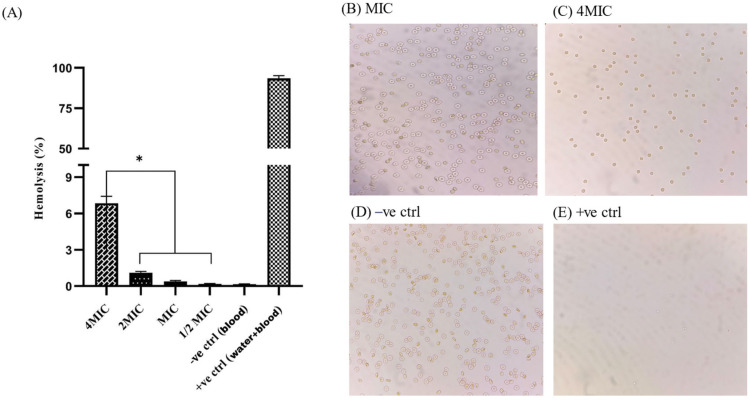
The hemocompatibility assay showed a statistically significant difference in percentage hemolysis between 4MIC and lower values (2MIC, MIC, 1/2MIC) (**A**). Microscopic slides were prepared from different wells of a 96-well plate to examine the morphology of RBCs after hydrogel treatment. At the MIC value, RBCs were intact and in good numbers (**B**), similarly to the negative control (**D**). RBCs were decreased in number when treated with 4MIC value of hydrogel (**C**), and were completely hemolyzed in the positive control (**E**) * represent the statistical significant difference at 0.05. RBCs morphology was observed under light microscope at 40×.

The hemocompatibility assay was performed in a 96-well plate, and absorbance was measured at 450 nm using a microplate ELISA reader. At the MIC value (29.30 µg/mL), no visible agglutination was observed, and a negligible level of hemolysis (0.385%) was detected. A statistically significant difference (*p* < 0.05) was observed for percentage hemolysis at 4MIC and lower values (2MIC, MIC, 1/2MIC). Microscopic slides were prepared from the 96-well plate to examine the morphology of the red blood cells (RBCs). The RBCs were intact and present in good numbers at 2MIC and MIC values. Controls were run to ensure the reliability of the assays (Figure 11).

### 2.12. Biofilm Assay

**Figure 12:**
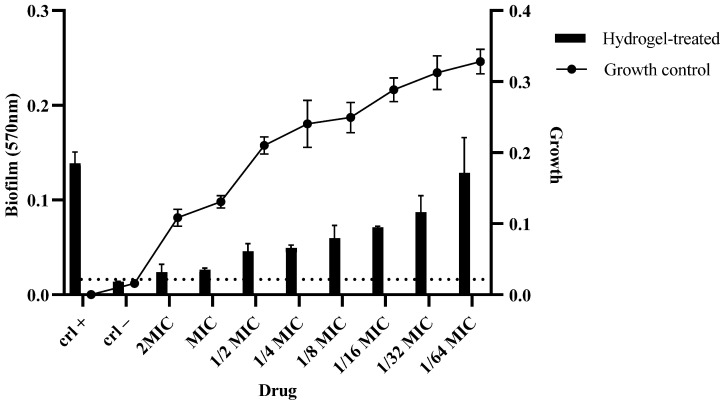
A biofilm assay was performed to assess the ability of the hydrogel to degrade the biofilms formed by *C. auris*. The strains were allowed to form biofilms overnight in a 96-well plate, and the hydrogel was added to determine the degradation efficacy. The experiment was performed in triplicate. Dotted line indicate the background reading of hydrogel.

The impact of different concentrations of the hydrogel on the biofilm formation and growth of MDR *C. auris* was assessed. At concentrations of 2MIC, MIC, and 1/2MIC, biofilm formation was significantly reduced in comparison to the growth control. As the concentration decreased from 1/4 to 1/64MIC, biofilm formation gradually increased (Figure 12).

### 2.13. In Silico Analysis

#### 2.13.1. Molecular Properties and Drug Likeness

The in silico analysis focused on the potential of CS, MAA, and PEG as drug candidates, indicating their hydrophilicity, topological polar surface area (TPSA), hydrogen bond donors (HBDs), hydrogen bond acceptors (HBAs), atomic weight, and rotatable bonds. The results show that CS has the highest TPSA, whereas MAA has the highest bioavailability score, suggesting better oral bioavailability. PEG has the most rotatable bonds, suggesting a higher molecular flexibility. All compounds score 0 for bioassay interference and comply with Lipinski and Veber rules, indicating good drug-like properties (Table 2).

#### 2.13.2. In Silico Interaction with Cytochrome Enzymes

In silico analysis of CS, MAA, and PEG with cytochrome P450 enzymes revealed that none of these compounds inhibited these enzymes. This lack of inhibitory activity suggests that these compounds are unlikely to interfere with metabolic processes mediated by these enzymes. This results in minimal drug–drug interactions and a predictable metabolic profile, which is crucial for understanding their pharmacokinetics. This positive outlook on the metabolic interactions of CS, MAA, and PEG supports their potential as safe and effective drug candidates, without significant concerns regarding cytochrome P450 enzyme inhibition (Table 3).

#### 2.13.3. Toxicity Prediction

Table 4 shows the median lethal dose (LD_50_), toxicity class, and potential toxic effects. CS has moderate acute toxicity, MAA has high acute toxicity, and PEG has low acute toxicity. Hepatotoxicity and neurotoxicity were predicted to be inactive. For CS, respiratory toxicity was predicted to be active (0.57), whereas for MAA and PEG, it was predicted to be inactive. The results provide an understanding of the safety profiles of CS, MAA, and PEG, aiding their assessment as potential drug candidates (Table 4).

#### 2.13.4. Nuclear Receptor Signaling Pathway Toxicity Prediction

In terms of the toxicity of CS, MAA, and PEG to nuclear receptor signaling pathways, it was predicted that the compounds are inactive in the AhR pathway and in the AR-LBD, aromatase, and ER-LBD domains. They do not inhibit aromatase, an enzyme critical for estrogen biosynthesis. These compounds are unlikely to interfere with the ligand-binding domain of the androgen receptor. These pathways are crucial for the regulation of gene expression, and can influence the toxicity and therapeutic efficacy of drug candidates (Table 5).

## 3. Discussion

Candidemia caused by *C. auris* is a growing concern for healthcare practitioners and researchers. Some *C. auris* isolates have shown pan-resistance to available antifungal drugs [31]. Invasive fungal infections are difficult to treat, and are gradually increasing in number, due to improper diagnosis and malpractice of antifungal drugs in low- and middle-income countries (LMIC).

The current study was designed to formulate a hydrogel against resistant strains of *C. auris.* The hydrogel was synthesized from CS, PEG, and MAA, and characterization was performed for morphology, swelling, degradation, and drug release profile. The hydrodynamic radius (554.7 ± 90.1 nm) of our formulation aligns with previous studies suggesting that hydrogels with a hydrodynamic radius between 200 and 600 nm have better bioavailability [32]. The FTIR spectrum of the hydrogel exhibited characteristic peaks that confirmed the presence of CS, PEG, and MAA functional groups within the synthesized hydrogel. The OH stretch at 3350 cm^−^^1^, along with the NH stretch at 3273 cm^−^^1^ and multiple CO stretch bands, validates effective crosslinking between the components, which is supported by similar findings [33]. The FT-IR spectra of CS [34], PEG [35], and MAA [36] have already been reported in the literature. Previous studies have reported that similar functional group bonds exist in CS-based hydrogels, revealing that PEG incorporation enhances the hydrogels’ solubility and mechanical characteristics [37]. During the current study, the results from TGA indicated that the hydrogel lost approximately 50% of its weight upon reaching a temperature of 460 °C, indicating its intended suitability during the sterilization process in biomedical applications [38,39]. CS-based hydrogels maintain their structural integrity at higher temperatures, as supported by research findings that support their use in applications such as drug delivery and wound healing [40]. Conversely, CS-based hydrogels have been found to be unstable at higher temperatures [41]. The preparation methods for hydrogels and the ratios of their components result in variations in their properties. Achieving suitable thermal and mechanical properties requires precise adjustments in formulation conditions designed to meet specific application needs.

Our formulation showed maximum swelling and drug release at physiological pH. The formulation exhibited non-Fickian diffusion of fluconazole. In hydrogels, which are hydrophilic polymer networks extensively used for controlled drug delivery, non-Fickian behavior is often observed, due to the dynamic nature of hydrogel swelling and the relative rates of solvent penetration and polymer relaxation [42]. When 0.45 < n < 0.89, this suggests a coupling between diffusion and polymer relaxation for drug release [43]. Different studies have found that crosslinking agents and polymers affect the swelling, degradation, and drug release profile of hydrogels [44]. Hydrogel swelling and degradation depend on the type of loaded drug [45,46] and its structural composition, such as CS, collagen, PEG, gelatin, and alginate [47,48]. In one study, the addition of the crosslinker genipin to a CS-based hydrogel was shown to alter the drug release kinetics of the hydrogel [49]. Similarly, the loading of nanoparticles was found to affect the swelling profile of the hydrogel [50]. Biomedical applications can benefit from an understanding of how pH, temperature, and environmental conditions affect swelling. Understanding these interactions can help in designing biomedical-specific CS-based hydrogels with optimal therapeutic outcomes.

In the current study, after treatment with the hydrogel, yeast cells showed diffuse irregular morphology upon staining with CFW. These findings suggest that the interaction of the hydrogel with yeast cell membranes leads to increased permeability and, eventually, cell death [51]. CS, a cationic compound, has broad antifungal properties, owing to its ability to interact with negatively charged fungal cell membranes. This is particularly relevant in the case of *C. auris*, where the ability of the hydrogel to compromise cell membrane integrity makes both sensitive and resistant strains susceptible to its antifungal effects [16,52,53]. The molecular weight and degree of deacetylation of CS also influence its antifungal activity. Low-molecular-weight CS penetrates fungal cells more effectively, inhibiting vital cellular processes such as DNA and RNA synthesis [54,55]. The presence of MAA in the hydrogel formulation may have enhanced its antifungal efficacy. The ability of the hydrogel to form a protective layer inhibits biofilm formation, thereby addressing the challenge of treating infections caused by resistant strains [56]. Our findings are supported by similar studies in which antifungal agents were found to disrupt the membranes of fungal cells [57,58]. CS has been found to possess anti-quorum-sensing properties and stop the maturation of biofilm formation [59]. CS also disrupts the extracellular matrix of biofilm through electrostatic interactions [60]. Research has demonstrated the potential of CS-based hydrogels for antifungal applications. Chemically cross-linked hydrogels showed antifungal activity comparable to that of the physical gels. New hydrogels based on piperonyl-imino-CS derivatives demonstrated sustained antifungal drug release and promising antifungal activity against Candida strains [56]. The effect of the freeze–thaw technique on alginate/CS glutamate gels loaded with posaconazole demonstrated antifungal activity [61]. An advanced biomaterial agent made from a CS/poloxamer 407-based thermosensitive hydrogel containing biosynthesized AgNPs demonstrated broad-spectrum antimicrobial activity against multidrug-resistant clinical isolates [62]. Cu-CS nanocomposite hydrogels demonstrated strong antifungal activity against aflatoxigenic Aspergillus flavus [63]. In a different context, prepared antibacterial polyvinyl alcohol/PEG/CS hydrogels containing silver chloride nanoparticles showed a high bacteriostatic rate, which could potentially extend to antifungal activity, highlighting the versatility of hydrogel systems for combating various pathogens [64].

The current study shows the hemocompatibility and antibiofilm properties of hydrogels, which indicates the potential of formulation for injectable systems and wound healing. CS-based hydrogels are being studied for use in wound dressings [65,66,67,68]. They have also been shown to prevent hemorrhage during wound healing [69]. In a similar study, a CS-PEG-based hydrogel showed increased plasma albumin absorption and hemocompatibility [7]. Similarly, chitin-CS composite fibers showed negligible hemolysis and hemocompatibility [70]. Studies have demonstrated the antibiofilm properties of CS-PEG hydrogels against various fungi. These hydrogels inhibit components of the SAGA complex and efflux transporter genes, demonstrating their potential for combating fungal infections [71]. Physically cross-linked hydrogels have also shown inhibitory activity, owing to interactions between the CS amino groups and the negatively charged fungal membrane [72].

Additionally, CS-based hydrogels have been investigated for the sustained release of antifungal drugs, and have shown promising results against Candida strains [56]. In silico analysis of the CS-PEG-MAA hydrogel showed minimal toxicity and low drug–drug interactions, making it a suitable candidate for biomedical applications. Our findings are supported by the fact that CS shares structural similarities with GAG and hyaluronic acid, exhibiting high biocompatibility, low toxicity, and non-immunogenic activity. Its tunable properties [73], hemocompatibility [70], and versatility in wound dressing materials [38] support its potential in drug delivery systems and wound-healing applications.

Considering the increasing resistance of *C. auris* to commonly available antifungal drugs, and their side effects, limited availability in underdeveloped countries, high cost of treatment, and high mortality rate, alternative therapeutics are required. The increasing trend of drug resistance limits therapeutic options. Our formulation was designed to address these challenges. Hydrogels have been found to be an effective treatment strategy in various clinical presentations. The current formulation has strong antifungal activity, with a satisfactory swelling and drug release profile, good bioavailability, antibiofilm properties, and low toxicity in in silico analysis. The formulation was found to be hemocompatible, and can be tested in ex vivo and in vivo studies. The formulation can be modified according to the desired conditions and pH, and can be applied to wound dressings and skin infections. The above discussion and the limited number of studies make the formulation a good choice for this cause.

## 4. Materials and Methods

### 4.1. Materials

Clinical isolates of *C. auris* were provided by the Jinnah Postgraduate Medical Center (JPMC), Karachi, Pakistan. Low-molecular-weight CS (MW: 50–190 KDa, Viscosity: 20–300 cP, soluble in dilute aqueous acid, deacetylation ≥75%), PEG-8000 (MW: 7000–9000 Da), MAA (MW: 86.09 Da, potency: 1320 mg/kg LD_50_, pH: 2.0–2.2 (20 °C, 100 g/L in H_2_O), 1 M Tris-HCl (pH: 6.5), Fluconazole, Amphotericin B, and crystal violet were obtained from Sigma-Aldrich.

### 4.2. Isolation and Identification of C. auris

The glycerol stock of the strains was cultured on Sabouraud Dextrose Agar (SDA) and CHROMagar (Condalab, Madrid, Spain). Identification was later confirmed using API 20C aux and matrix-assisted laser desorption ionization time-of-flight mass spectrometry (MALDI-TOF MS, Biomerieux, Lyon, France).

### 4.3. Antifungal Susceptibility Testing

Antifungal susceptibility testing was performed using an E-test plastic strip, and broth dilution methods were used to analyze the MIC, according to the Centers for Disease Control and Prevention (CDC) guidelines (M27, Ed 4). The isolate showing an MIC of ≥2 µg/mL for Amphotericin B was considered resistant, and was used for assessment of the antifungal and antibiofilm activity of our hydrogel [27].

### 4.4. Synthesis of Hydrogel

The hydrogel was prepared by gradually dissolving CS in an acidic solution, followed by addition of PEG and MAA using a magnetic stirrer for homogenization. A 1 g sample of CS was dissolved gradually in 50 mL of 0.1 N HCl at 37 °C, and was left for 8 h, with continuous stirring (250 rpm) with magnetic stirrer. A 670 mg/mL sample of PEG-8000 was dissolved in distilled water at 200 rpm and 20 °C. A 15% MAA solution was then prepared and dissolved in water. CS, PEG, and MAA were mixed in a volumetric ratio of 2:1:1 for 1 h at 200 rpm, using a magnetic stirrer (Thermo Fisher Scientific, Waltham, MA, USA). The prepared hydrogels were stored at 4 °C.

### 4.5. Characterization of Hydrogel

#### 4.5.1. Zeta Potential and PDI Measurement

The zeta potential, mean particle size, and polydispersity index (PDI) were determined using a zeta-sizing instrument, based on dynamic light scattering (DLS) (Zeta Sizer Nano, Malvern Instruments, Malvern, UK).

#### 4.5.2. Morphological Studies

The hydrogel samples were dried overnight in a hot-air oven at 45 °C, and characterized using scanning electron microscopy (FE-SEM; TESCAN Vega LMU, Huntingdon, UK). High-resolution images were produced by analyzing the surface topography, porosity, and structural features at resolutions of 10, 5, and 1 µm [23].

#### 4.5.3. Fourier Transform Infrared (FT-IR) Spectroscopy

FTIR spectroscopy of the hydrogel sample was performed using an A2 Technologies portable attenuated reflectance (ATR) Fourier Transform Infrared Spectroscopy (ATRS-FTIR) spectrometer (New Orleans, LA, USA). The dried sample (1 mg) was used for functional group analysis [28].

#### 4.5.4. Thermogravimetric Analysis

TGA was performed to investigate the thermal stability and decomposition behavior of the hydrogels. A small amount of hydrogel was inserted into the TGA instrument (Perkin Elmer, Springfield, IL, USA), which heated the samples at a constant rate, from room temperature to a maximum temperature of 800 °C (10–20 °C/min) [17]. The instrument continuously measured and recorded the weight change of the sample as a function of temperature, generating a thermogravimetric (TGA) curve.

### 4.6. Swelling and Degradation Assay of Hydrogel

The swelling property [29] of the hydrogel was measured in buffers of different pH values, including acetate buffer (pH 4.0), phosphate-buffered saline (pH 7.4), and borate buffer (pH 10). The degradation assay was performed in phosphate-buffered saline at pH 7.4 ± 1, and continued until the hydrogel was completely degraded and no physical mass remained. All experiments were performed in triplicate.

The swelling percentage was calculated by following equation:

*Swelling percentage = (Ws − Wd)/Wd* × 100 (1)

‘*Ws*’ = the weight of the swollen hydrogel, and ‘*Wd*’ = the weight of the dry hydrogel.

For the degradation assay, the percentage of weight loss was calculated using the following equation:

*Weight loss percentage = (Wi − Wt)/Wi* × 100 (2)

‘*Wi*’ = the initial weight, and ‘*Wt*’ = the remaining weight of the hydrogel.

### 4.7. Drug Entrapment Efficiency (%DEE) of Hydrogel

The entrapment efficiency of the hydrogel was evaluated to determine the drug-holding and release capacities of the CS-PEG network [30]. Fluconazole, an antifungal drug, was loaded onto the hydrogel at a concentration of 32 µg/mL. The hydrogel was immersed in three different pH conditions: acetate buffer (pH 4.0), phosphate-buffered saline (pH 7.4), and borate buffer (pH 10). The drug-loaded hydrogel was continuously stirred at 150 rpm at 37 °C. A 1 mL volume of the solution was collected periodically, and absorbance was measured at 250 nm (Thermo Scientific 50 UV-Vis, Waltham, MA, USA). After each reading, fresh buffer was added to maintain a constant volume. The experiments were performed in triplicate. The drug entrapment efficiency (%DEE) was calculated using the following equation:

*%DEE = (Total Drug conc. − Supernatant Drug conc.)/(Total Drug conc.)* × 100 (3)

Drug release kinetics models were constructed using following equations [24]:

Zero-order:

*Mt = Mo + Ko t* (4)

First-order:

*logC = logCo* − *Kt*/2.303 (5)

Korsmeyer–Peppas model:

*lnMt/Mo = n lnt + lnK* (6)

### 4.8. Antifungal Activity of Hydrogel

The antifungal activity of the hydrogel was initially determined using a disk diffusion assay, followed by determination of its minimum inhibitory concentration (MIC). All dilutions and protocols were performed (in triplicate) according to CLSI guidelines (M27, Edition 4) for antifungal susceptibility testing of yeasts [31]. The antifungal activity was also evaluated against sensitive isolates to assess the comparative efficacy of the hydrogel. The absorbance of the 96-well plates was measured at 600 nm using a VersaMax ELISA plate reader (StakMax^®^, San Jose, CA, USA).

### 4.9. Calcofluor White Staining and Fluorescent Microscopy of C. auris Cells

To assess the morphology of yeast cells and the killing mechanism of the hydrogel, fluorescence microscopy was performed, and images were taken with a Zeiss M2 upright microscope (Neissie, San Antonio, TX, USA). The MDR strain of *C. auris* was freshly grown in 10 mL of YPD medium for 24 h. After 24 h, the cells were washed properly with PBS, and were adjusted to proper concentration (1 × 10^6^). The cells were treated with the MIC value (≈30 µg/mL) of the hydrogel, and incubated for 24 h at 37 °C. The PBS control was used for drug treatment. After 24 h, the cells were washed three times with PBS, incubated with 20 µg/mL calcofluor white (CFW), and kept in the dark for 1 h. After incubation and washing with PBS 3 times, the pellet was resuspended, and a drop was added to the slides, followed by mounting media containing DAPI (Invitrogen, Waltham, MA, USA) and cover slips.

### 4.10. Hemocompatibility Assay of Hydrogel

A hemocompatibility assay was performed to assess the compatibility of the hydrogel with red blood cells (RBCs) and for the in vivo studies [74]. Human blood samples were obtained from the healthy volunteers. The RBCs were washed thrice with normal saline, and a 5% suspension of RBCs was prepared. The hydrogel was serially diluted in a 96-well microplate, and a suspension (100 µL) of RBCs was added. The 96-microwell plate was incubated for 04 h to observe any visible agglutination or hemolysis. The absorbance of the wells was measured at a wavelength of 490 nm using a microplate ELISA reader (StakMax^®^, California, USA). Microscopic slides were prepared from each well to observe the RBC morphology. The following formula was used to calculate the percentage of hemolysis caused by the hydrogel:

% *Hemolysis = OD_Tes_t − OD_Neg_/OD_Pos_ − OD_Neg_* (7)

OD represents the absorbance (optical density) at 490 nm; *OD_Neg_/OD_Pos_* of negative and positive control.

### 4.11. Biofilm Assay

The crystal violet biofilm assay was performed in a 96-well plate. The MDR *C. auris* strains were cultured overnight in YPD medium at 30 °C and 200 rpm. Once the overnight culture was complete, the cells were washed with dH_2_O and diluted to a concentration of 1 × 10^6^ cells/mL in 20% serum + dH_2_O. To initiate biofilm formation, 200 µL of the cell suspension was added to each well of a 96-well plate, and incubated at 37 °C for 1.5 h. Following incubation, the cells were gently washed thrice with PBS and left to dry overnight. Various dilutions of the hydrogels were prepared and added to the corresponding wells. The plates were then incubated overnight at 37 °C. After incubation, 100 µL of 0.04% crystal violet solution was added to each well. After a 20 min incubation period, the plate was washed under running water. Ethanol (100%, 100 µL) was then added to each well. The resulting supernatant was transferred to a fresh 96-well plate, and the OD was measured at 570 nm using a VersaMax™ Tunable Microplate Reader. Growth controls were used to ensure the reliability of the experiment, and the absorbance of the growth control was measured at 600 nm [56].

### 4.12. In Silico Analysis

This study analyzed the molecular properties and drug likeness of CS, PEG, and MAA based on their interactions with cytochrome P450 enzymes, and their toxicity was predicted using ProTox-II 3.0. This study also predicted the toxicity related to nuclear receptor signaling pathways.

### 4.13. Data Analysis

Microsoft Excel was used for data storage, organization, and analysis. Drug release kinetic models were constructed using MS Excel. Graphpad Prism v9.0 was utilized to run a nonlinear regression model, and calculated the values by fitting the dose–response curves to a sigmoidal model.

## 5. Conclusions and Future Recommendations

The cationic hydrogel, with a hydrodynamic radius of 554.7 ± 90.1 nm and a charge of 15.16 ± 1.09 mV, is suggested to have compatibility for topical and in vivo usage, respectively. The SEM analysis revealed a porous structure suitable for drug loading and release. The drug loading release mechanism showed non-Fickian diffusion at three different pH values. The developed formulation showed effective antifungal (MIC = 29.30 ± 11.72 µg/mL) and antibiofilm properties against MDR *C. auris*, and was found to be hemocompatible, indicating potential for in vivo delivery systems. After staining with CFW, the hydrogel was found to interact with the cell wall, leading to increased permeability, and eventually, death of *C. auris*.

In silico analysis of CS, PEG, and MAA revealed drug likeliness, lower toxicity, and no inhibition of cytochrome P450 enzymes. The components were inactive against nuclear receptor signaling pathways. Future research focusing on in vivo and cell line studies could pave the way for better understanding of the formulation and development of injectable systems.

## Figures and Tables

**Table 1:** The drug release kinetics of the hydrogel at three different pH values.

pH	Release Medium	Zero-Order Kinetics		First-Order Kinetics		Korsemeyer–Peppas Model		Mechanism of Drug Release
		R^2^	K_0_	R^2^	K_1_	R^2^	*n*	
4.0	Acetate buffer	0.894	0.120	0.846	0.009	0.967	0.54	Non-Fickian diffusion
7.4	Phosphate-buffered saline (PBS)	0.958	0.113	0.942	0.006	0.974	0.75	Non-Fickian diffusion
10	Borate buffer	0.894	0.122	0.854	0.008	0.978	0.59	Non-Fickian diffusion

**Table 2:** In silico molecular properties and drug likeness of CS, methacrylic acid (MAA), and PEG.

Tested Items	CS	PEG	MAA
Log P	0.88	−1.03	0.65
TPSA (Å)	56.79	40.46	37.30
HBDs	1	2	1
HBAs	5	2	2
MW (g/mol)	184.1	62.07	86.1
nRT	4	1	1
PAINS	0	0	0
Bioavailability score	0.55	0.55	0.85
Synthetic accessibility	3.14	1.00	1.01
Lepinski rule	Yes (0)	Yes (0)	Yes (0)
Veber rule	Yes	Yes	Yes

**Table 3:** In silico molecular properties and interactions of CS, MAA, and PEG with cytochrome enzymes.

Tested Items	CS	PEG	MAA
CYP2C19 inhibitor	No	No	No
CYP3A4 inhibitor	No	No	No
CYP1A2 inhibitor	No	No	No
CYP2C9 inhibitor	No	No	No
CYP2D6 inhibitor	No	No	No

**Table 4:** In silico toxicity prediction of CS, MAA, and PEG.

Tested Items	CS	PEG	MAA
Predicted LD_50_ (mg/kg)	1000	4700	118
Toxicity class	4	5	3
Neurotoxicity	Inactive (0.50)	Inactive (0.90)	Inactive (0.62)
Hepatotoxicity	Inactive (0.77)	Inactive (0.80)	Inactive (0.65)
Carcinogenicity	Inactive (0.58)	Inactive (0.79)	Inactive (0.78)
Respiratory toxicity	Active (0.57)	Inactive (0.57)	Inactive (0.87)
Mutagenicity	Inactive (0.64)	Inactive (0.89)	Inactive (0.89)

**Table 5:** In silico nuclear receptor signaling pathway toxicity prediction.

Tested Items	CS	PEG	MAA
AhR	Inactive (0.96)	Inactive (0.97)	Inactive (1.0)
AR	Inactive (0.98)	Inactive (0.98)	Inactive (1.0)
AR-LBD	Inactive (0.98)	Inactive (0.96)	Inactive (0.99)
Aromatase	Inactive (0.94)	Inactive (1.0)	Inactive (1.0)
ER	Inactive (0.89)	Inactive (0.91)	Inactive (0.90)
ER-LBD	Inactive 0.97)	Inactive (0.98)	Inactive (1.0)
PPAR-Gamma	Inactive (0.95)	Inactive (0.95)	Inactive (0.99)

## Data Availability

The experimental data have been presented in the paper.

## Supplementary Materials

The following supporting information can be downloaded at: https://www.mdpi.com/article/10.3390/ph18040506/s1, Figure S1: The drug release kinetics of hydrogel, showing zero-order model, first-order model, and Korsemeyer–Peppas model at pH 4.0 (a, b, c) and pH 7.4 (d, e, f), respectively. The Korsemeyer–Peppas model was the best-fit model, showing a non-Fickian diffusion mechanism. Figure S2: The drug release kinetics of the hydrogel, showing the zero-order model (a), first-order model (b), and Korsemeyer–Peppas model (c) at pH 10.0. The Korsemeyer–Peppas model was the best-fit model, showing a non-Fickian diffusion mechanism. Figure S3: Swelling and degradation of the hydrogel at physiological pH.

## Abbreviations

MIC, minimum inhibitory concentration; MDR, multidrug resistance; CLSI, Clinical Laboratory Standard Institute; CFW, calcofluor white; CS, chitosan; PEG, polyethylene glycol; MAA, methacrylic acid.
