# Supplementary material for: Synthesis and Evaluation of a Chitosan-Based Cationic Hydrogel with Strong Antifungal and Antibiofilm Activities Against Clinical Isolates of Candida auris
Source: Pharmaceuticals (Basel). 2025 Mar 31;18(4):506. doi: 10.3390/ph18040506 (PMC12030561; doi:10.3390/ph18040506)
Supplement: Supplementary file 1 [file pharmaceuticals-18-00506-s001.zip › pharmaceuticals-3499712-supplementary.pdf]

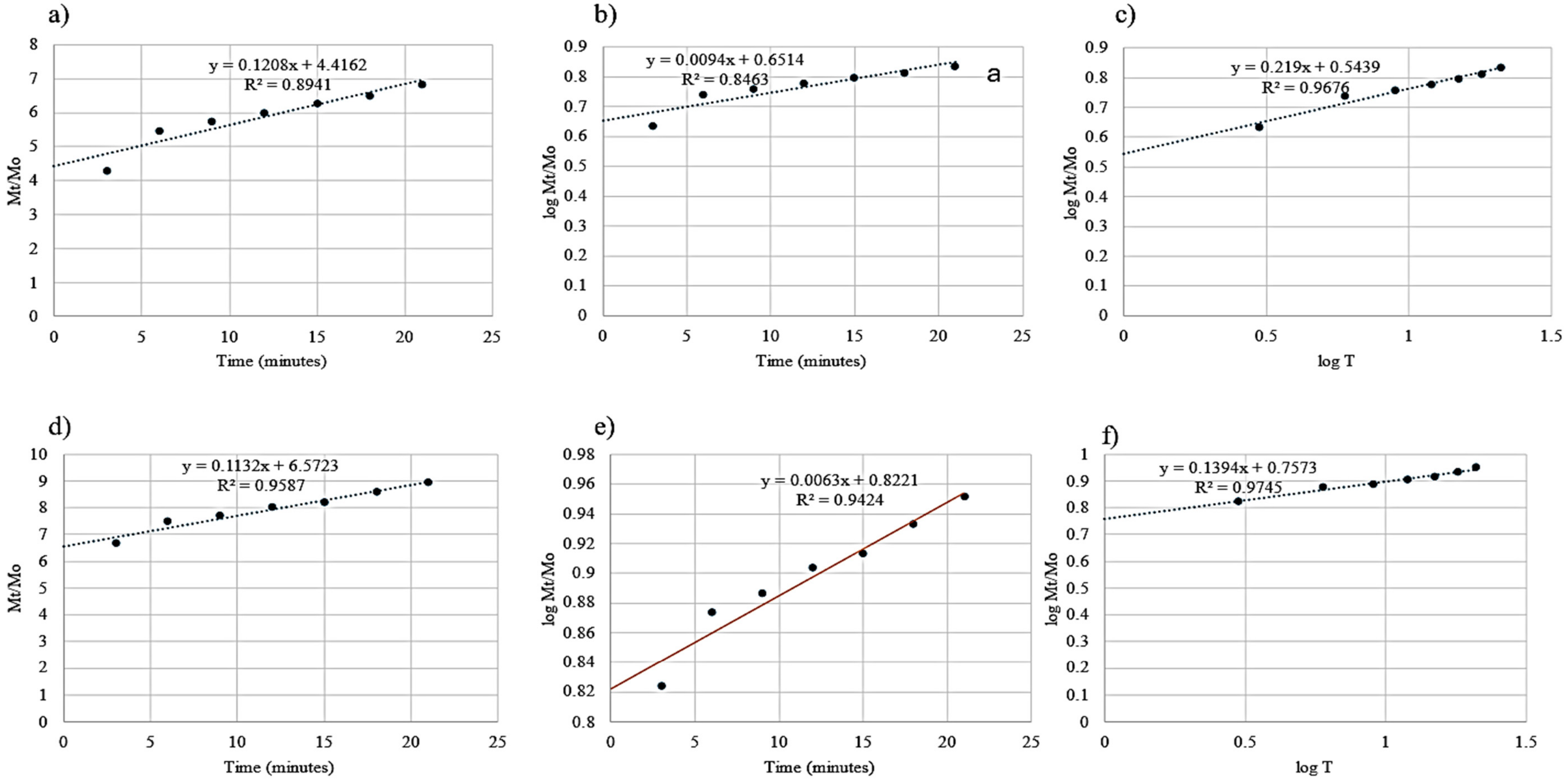

Figure S1: Drug release kinetics of hydrogel showing zero order, first order and Korsemeyer–Peppas Model at pH 4.0 (a, b, c) and at pH 7.4 (d, e, f) respectively. Korsemeyer–Peppas Model was the best fit model showing non-Fickian diffusion mechanism.

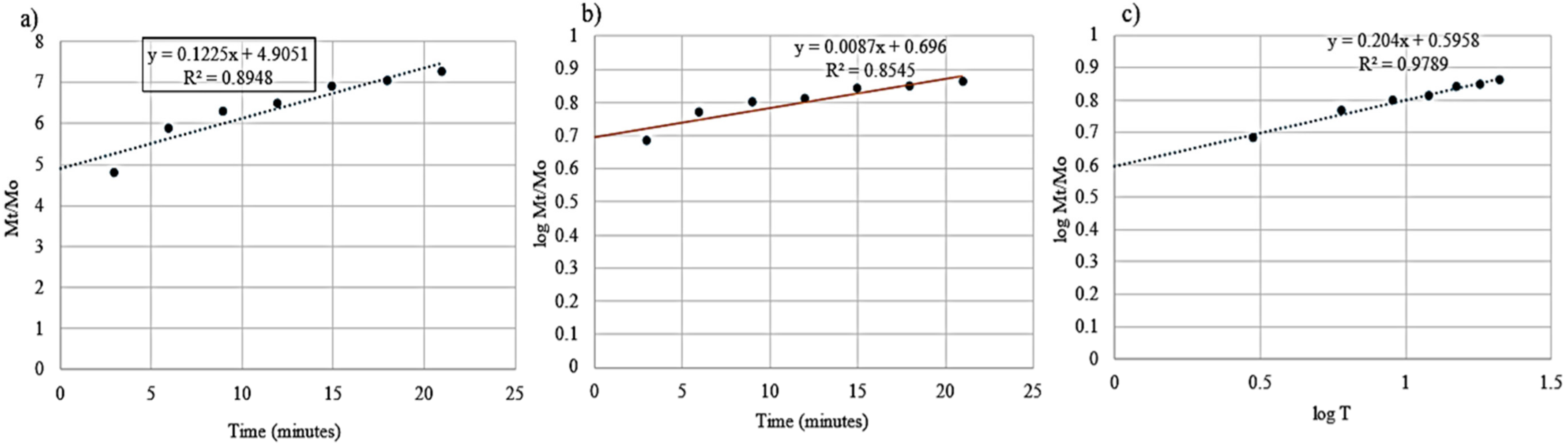

Figure S2: Drug release kinetics of hydrogel showing zero order (a), first order (b) and Korsemeyer–Peppas Model (c) at pH 10.0. Korsemeyer–Peppas Model was best fit model showing non-Fickian diffusion mechanism.

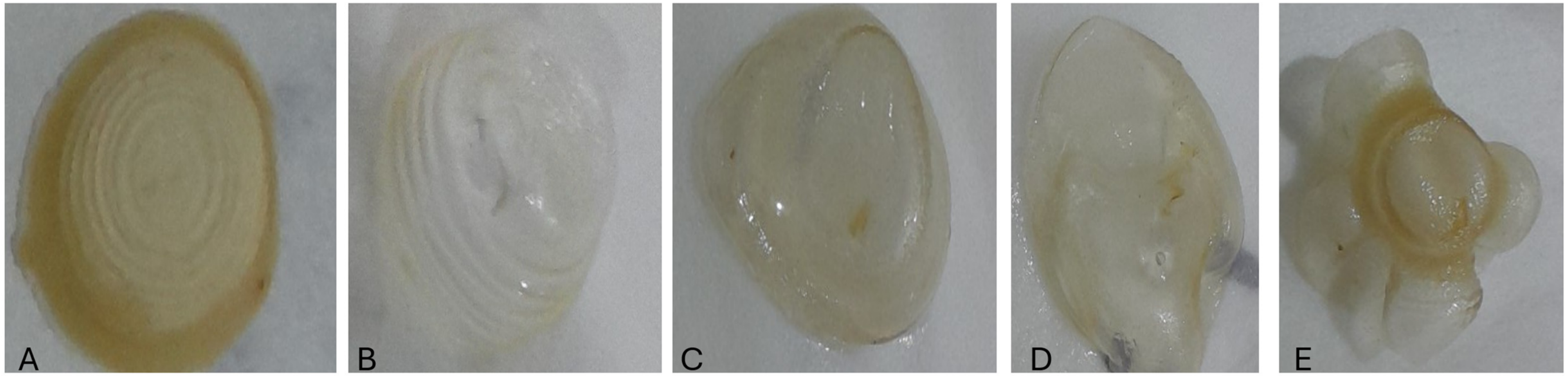

Figure S3: Imges of the hydrogel showing swelling then moving towards degradation. A) Dried hydrogel, B) Hydrogel start swelling at Physiological pH, and after maximum swelling (C), hydrogel start to shrink and degrade (D, E).
